# Vaccination coverage in rural Burkina Faso under the effects of COVID-19: evidence from a panel study in eight districts

**DOI:** 10.1186/s12913-023-10029-1

**Published:** 2023-09-21

**Authors:** Sarah Cooper, Frank Bicaba, Cheick Oumar Tiendrebeogo, Alice Bila, Abel Bicaba, Thomas Druetz

**Affiliations:** 1 Department of Social and Preventive Medicine, School of Public Health, University of Montreal, Montreal, QC Canada; 2 Société d’Études et de Recherches en Santé Publique, Ouagadougou, Burkina Faso; 3 Sciences de la Vie et de la Santé, University Aix-Marseille, Marseille, France; 4 Centre de recherche en santé publique, Montreal, QC Canada; 5 Department of Tropical Medicine, Tulane University School of Public Health and Tropical Medicine, New Orleans, LA USA

**Keywords:** Routine vaccination, Immunization, Children, Burkina Faso, COVID19, Vaccination coverage

## Abstract

**Background:**

Improving infant immunization completion and promoting equitable vaccination coverage are crucial to reducing global under-5 childhood mortality. Although there have been hypotheses that the impact of the COVID-19 pandemic would decrease the delivery of health services and immunization campaigns in low- and middle-income countries, the available evidence is still inconclusive. We conducted a study in rural Burkina Faso to assess changes in vaccination coverage during the pandemic. A secondary objective was to examine long-term trends in vaccination coverage throughout 2010–2021.

**Methods:**

Using a quasi-experimental approach, we conducted three rounds of surveys (2019, 2020, 2021) in rural Burkina Faso that we pooled with two previous rounds of demographic and household surveys (2010, 2015) to assess trends in vaccination coverage. The study population comprised infants aged 0–13 months from a sample of 325 households randomly selected in eight districts (n = 736). We assessed vaccination coverage by directly observing the infants’ vaccination booklet. Effects of the pandemic on infant vaccination completion were analyzed using multi-level logistic regression models with random intercepts at the household and district levels.

**Results:**

A total of 736 child-year observations were included in the analysis. The proportion of children with age-appropriate complete vaccination was 69.76% in 2010, 55.38% in 2015, 50.47% in 2019–2020, and 64.75% in 2021. Analyses assessing changes in age-appropriate full-vaccination coverage before and during the pandemic show a significant increase (OR: 1.8, 95% CI: 1.14–2.85). Our models also confirmed the presence of heterogeneity in full vaccination between health administrative districts. The pandemic could have increased inequities in infant vaccination completion between these districts. The analyses suggest no disruption in age-appropriate full vaccination due to COVID-19. Our findings from our sensitivity analyses to examine trends since 2010 did not show any steady trends.

**Conclusion:**

Our findings in Burkina Faso do not support the predicted detrimental effects of COVID-19 on the immunization schedule for infants in low- and middle-income countries. Analyses comparing 2019 and 2021 show an improvement in age-appropriate full vaccination. Regardless of achieving and sustaining vaccination coverage levels in Burkina Faso, this should remain a priority for health systems and political agendas.

**Supplementary Information:**

The online version contains supplementary material available at 10.1186/s12913-023-10029-1.

## Contribution to the literature

Our study reveals an increase in vaccination coverage during the pandemic in Burkina Faso, contrary to prior research that reported or predicted decreases in routine immunization coverage. This cautions for a more nuanced understanding of the COVID-19 pandemic’s effects.

Vaccination coverage varies widely and increasingly between districts. The increase in the number of vaccines and supply difficulties in some areas during the pandemic have potentially contributed to this.

Policy- and decision-makers should prioritize considering local contextual factors that could influence vaccination coverage, as suggested by the significant disparities across districts in rural Burkina Faso.

**Supplementary Information:**

The online version contains supplementary material available at 10.1186/s12913-023-10029-1.

## Introduction

Since January 2020, the COVID-19 pandemic has been responsible for more than 6 million deaths worldwide, forcing many countries to take unprecedented measures to reduce its burden [1]. Efforts to reduce transmission have had indirect consequences on health systems, including disruption of supply chains, reduced mobility and access to services, closure of health facilities, and fear of visiting health facilities, all of which have likely worsened endemic disparities in access to healthcare services [2]. In many countries, logistical barriers and measures to reduce the risk of COVID-19 transmission have adversely affected door-to-door campaigns, and community-based programmatic efforts have been similarly disrupted [3, 4].

On March 26, 2020, the World Health Organization (WHO) called for a temporary suspension of mass vaccination campaigns, except for routine immunization programs in areas where risk of COVID-19 transmission was very low or negligible [5]. Since infant and child vaccinations are amongst the most successful public health interventions in low- and middle-income countries (LMICs) in terms of number of deaths averted per year [6], limiting these efforts raises serious public health concerns. Several countries have been faced with the public health dilemma of interrupting community-based immunization campaigns to control the transmission of COVID-19, increasing the risk of an epidemic rebound of childhood diseases normally kept under control by vaccinations. A similar situation was observed during previous disruptions of vaccination campaigns; notably, infant immunization coverage decreased during the Ebola outbreak in West Africa, which may have contributed to the increased incidence of measles in the region in the following years [7–10].

Following predictions that such an interruption in routine vaccination efforts would likely put ~ 80 million children at risk of vaccine-preventable diseases (such as poliomyelitis, measles, and diphtheria) and could result in a 45% increase in child mortality, the WHO and other organizations rapidly recommended to resume vaccination campaigns [11–13]. Despite this, the disruption caused by the pandemic has been associated with an estimated decline in global immunization coverage from 86% to 2019 to 81% in 2021, the lowest since 2009 [3, 14, 15]. Analyses of data from routine health information systems in several African, Asian, and South American countries have confirmed reductions in the number of children vaccinated at health facilities immediately after the onset of the pandemic [16–21].

These predictions and assessments from routine health information systems are likely biased for a number of reasons; for example, due to health personnel shortages and increased workload during the pandemic, data entry and transmission may have been reduced or interrupted. In addition, routine health information systems provide little information confirming whether infant immunization is actually administered, since vaccination data received during door-to-door visits of households are not always entered into health information systems. Further complicating matters, most studies have investigated only the short-term repercussions of the pandemic, focusing on the critical situation in 2020. For many LMICs, empirical, population-based evidence on vaccination coverage since the COVID-19 pandemic remains scarce.

Our study was designed to investigate changes in vaccination coverage in infants in rural Burkina Faso during the COVID-19 pandemic. While there have been reports of partial and transient disruptions of immunization services in the country [22], there is no empirical evidence on whether the pandemic has affected infant immunization coverage. Using panel data collected just before (2019–2020) and during (2021) the pandemic, this study’s primarily aim is to investigate the repercussions of the COVID-19 pandemic on infant vaccination coverage in Burkina Faso. The secondary objective is to examine long-term trends in vaccination coverage between 2010 and 2021, with the working hypothesis that the pandemic would decrease the coverage of age-appropriate complete vaccination and reverse the upward trends observed since 2010 [23]. This study is the first to offer an integrated perspective on the history of vaccination coverage and assess the lasting effects of COVID-19 on age-appropriate vaccination in infants in rural Burkina Faso.

## Methods

### Study setting and design

**Fig. 1:**
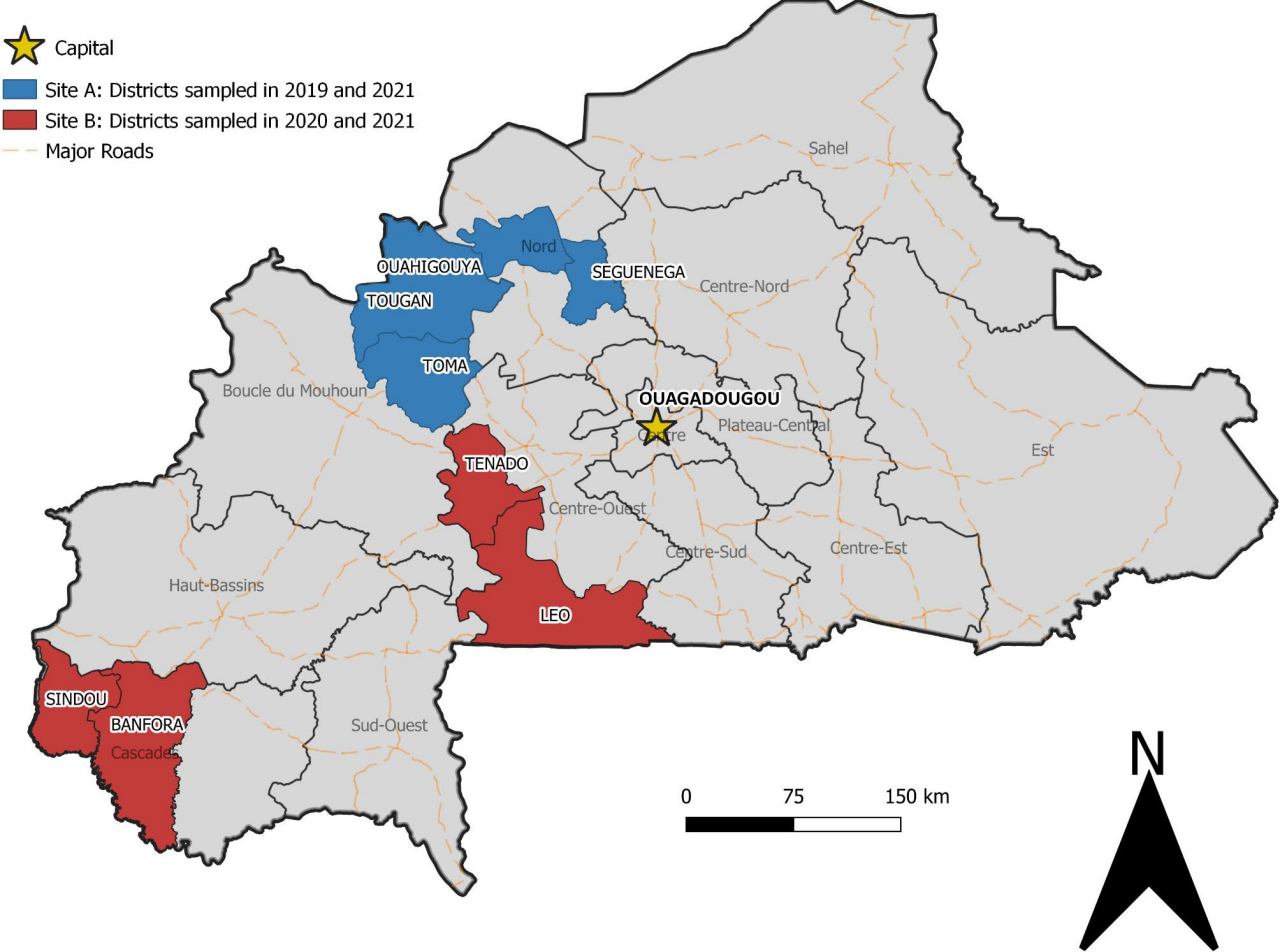
Map of study area. The figure was produced by the authors

This study was conducted in eight health districts in Burkina Faso that were purposively selected based on two criteria: they were considered relatively secure by the local health district authorities and the research team (i.e., were not affected by frequent terrorist attacks), and they were mostly rural and remote (> 100 km away from the capital, Ouagadougou). A map of the health districts selected for the study can be found in Fig. 1. The study area comprises a population of _~_2 million inhabitants for a territory of 27,161 km^2^, approximately one tenth of the total population and territory in Burkina Faso.

**Fig. 2:**
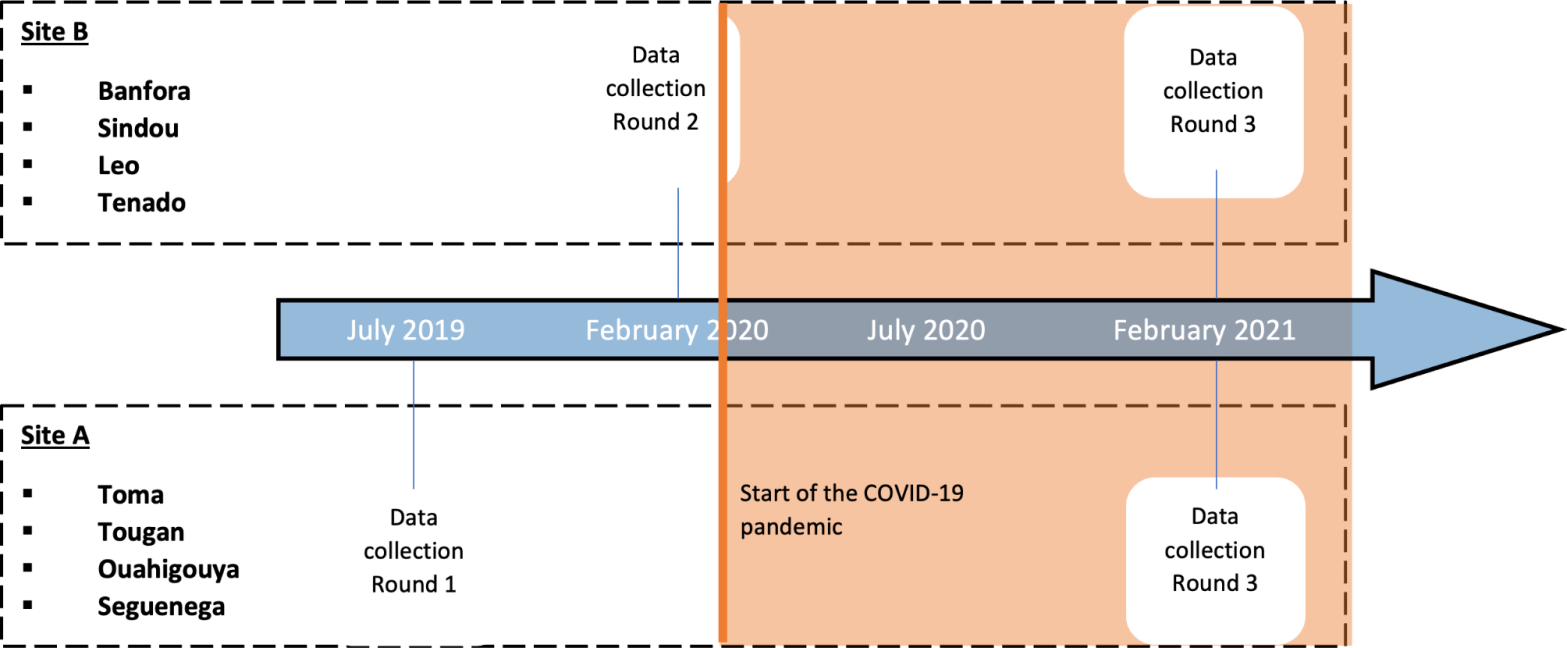
Timeline of the surveys

This study is a natural experiment that takes advantage of a research platform designed before the COVID-19 pandemic for a previous investigation by our group [2]. It uses primary data from repeated surveys conducted in a panel of 1,200 households. Each household was visited twice, once before the pandemic (July 2019 or February 2020) and once during the pandemic (February 2021). The four districts surveyed in July 2019 will be referred to as Site A, while the other four surveyed in February 2020 will be referred to as Site B (see Fig. 2). All districts were surveyed in February 2021. A quasi-experimental study was performed using a pre-post design to evaluate the repercussions of the pandemic on vaccination coverage in infants. More information about the study’s protocols and procedures is available in our previous publication [2].

### Recruitment of participants

The households in the panel were sampled using a stratified two-stage random sampling method, per the Demographic and Health Survey (DHS) protocol [24]. In the eight districts, 49 enumeration areas were first selected with probability proportional to size. In a second stage, 24 households per enumeration area were randomly selected from a complete list of households. Households without women of reproductive age (defined as 16–49 years) were not eligible. All households surveyed in 2019 or 2020 were systematically visited again in 2021. Losses to follow-up were not replaced.

All women aged 16–49 years and living in the sampled households were eligible and invited to participate in the study during the door-to-door survey. The nested cohort of women was open and dynamic (i.e., all women surveyed pre-pandemic remained eligible during the follow-up survey); therefore, in 2021, all women aged 16–50 years were invited to participate.

### Survey Procedures

A standardized sociodemographic questionnaire was administered individually to all participants. Questions were extracted from the DHS instruments (Supplementary file 1). The surveys gathered data on household features and participants’ main sociodemographic characteristics. A module of the questionnaire specifically targeted their children 0–13 months, documenting the specifics of care received at birth, recent episodes of illness, and their vaccination history. Detailed records of immunization (type of vaccine and date of administration) and date of birth were obtained from each child’s vaccination booklet. To avoid potential recall bias in rural areas, vaccinations self-reported by mothers were not recorded in cases where a child’s vaccination booklet was unavailable [25, 26]. More information about the survey procedures is available elsewhere [27].

### Outcomes and analyses

The unit of analysis is the child-year, i.e., the measurement observed in a child in a particular year [28]. A composite index of age-appropriate full vaccination was created based on Burkina Faso’s official immunization schedule. According to this schedule, children are to be immunized at birth against poliomyelitis and tuberculosis; receive three successive doses of the pentavalent (diphtheria, tetanus, pertussis, hepatitis B and *Haemophilus influenzae* type b), the pneumococcal conjugate, the poliomyelitis and the rotavirus vaccines at 8, 12 and 16 weeks, respectively; and the measles vaccine at 9 months (see Table 1). The outcome was binary, coded 1 if children were fully immunized for their age and 0 otherwise. Although it is recommended for children aged ∼10 months, the yellow fever vaccine was not routinely administered at the time of the survey due to a global shortage prompted by the 2016–2017 outbreak in the Democratic Republic of Congo and was therefore omitted from the present analyses [29].

**Table 1:** Infant immunization schedule in Burkina Faso

Contact	Time interval	Basic vaccines	Vaccines introduced in 2013
1	At birth	BCG, Polio 0	
2	8 weeks	Polio 1, Penta 1	Rota 1, Pneumo 1
3	12 weeks	Polio 2, Penta 2	Rota 2, Pneumo 2
4	16 weeks	Polio 3, Penta 3	Rota 3, Pneumo 3
5	9 months	Measles	

A statistical model was fitted using multiple logistic regression and the age-appropriate full vaccination indicator as dependent variable. A set of potential confounding variables were tested in the model: location and socioeconomic characteristics of the households (size, socioeconomic status, radio access, bicycle access, electricity access); education of the mothers; age of the mothers (in years); mothers’ occupation status; and the sex, birth order, birth setting, and age (in months) of the infant. The hierarchical structure of the data was taken into account by adding random intercepts at the household level and category of area (i.e., those surveyed in 2019 and 2021 and those surveyed in 2020 and 2021, Sites A and B, respectively), while specific trajectories of the districts were explored by adding random effects at the district level. The best fitting model was selected based on Akaike Information criterion values (i.e., a score that estimates the goodness of fit of a model based on predictive errors following parsimony procedures) [30]. Multicollinearity was ruled out by verifying that variance inflation factors did not exceed 4. Robust variance estimators were used throughout the analyses.

### Sensitivity analyses

Historical trends were determined by extracting data from the DHS conducted in the eight districts under study in 2010 and 2015. The same procedures described above were used to model the age-appropriate full vaccination for children < 14 months and calculate trends for 2010–2021. Further investigation was performed by repeating the analyses on another outcome: a composite index of age-appropriate full vaccination restricted to the vaccines that have been constantly administered since 2010 (vaccines against poliomyelitis, tuberculosis, measles, and the pentavalent, hereafter named “basic vaccines,“ and excluding the pneumococcal conjugate and rotavirus vaccines).

### Ethics

All participants recruited pre-pandemic provided written informed consent. As suggested and approved by the Health Research Ethics Committee in Burkina Faso and the Health Sciences Research Ethics Committee at University of Montreal, all participants recruited in 2021 provided informed consent verbally in order to reduce the risk of COVID-19 transmission. The questionnaire was administered individually in a secluded area to preserve participant confidentiality. Participants aged 16–17 years old were considered mature minors and consented as adults, as per national standards. All study procedures, including those for obtaining consent, were approved by the Health Sciences Research Ethics Committee at University of Montreal (Certificate #CERSES-20-146-D) and by the Health Research Ethics Committee in Burkina Faso (Deliberation #2018-6-075).

## Results

### Effects of COVID-19 pandemic on Vaccination Coverage

Analyses were restricted to 336 children < 14 months (n = 214 pre-pandemic, n = 122 during pandemic) for whom we were able to directly observe vaccination booklets (i.e., 68% of the total sample). Their main sociodemographic characteristics are described in Table 2. An average of 58% had age-appropriate full vaccination, but this coverage increased significantly during the pandemic, from 50% at baseline to 65% in 2021 (odds ratio = 1.8, 95% confidence interval [1.14–2.85]). Coverage ranged from 17 to 87% according to district and year.

**Fig. 3:**
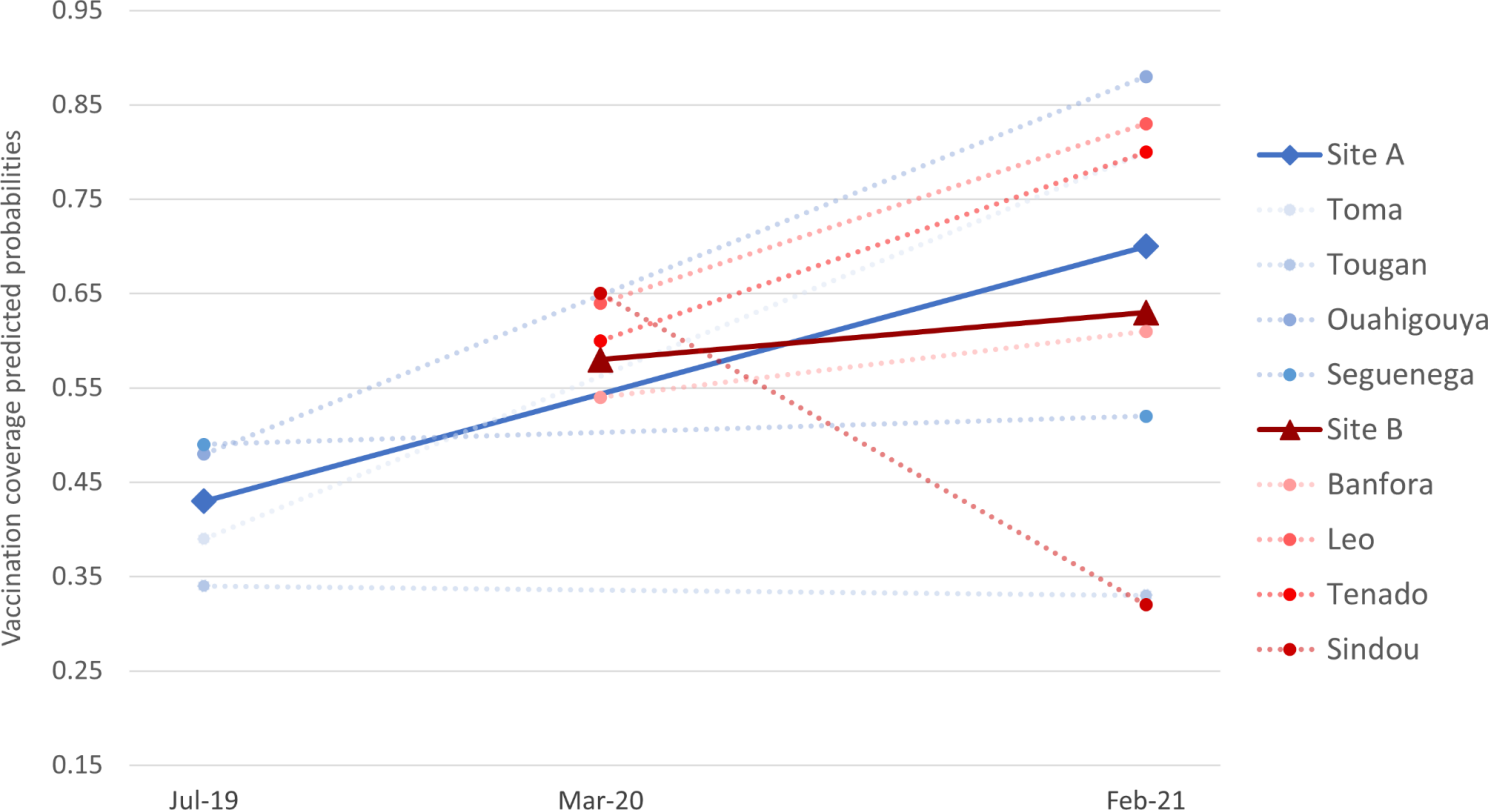
Infant’s predicted probabilities of having age-appropriate full vaccination in 2019, 2020 and 2021. Predicted probabilities are derived from a mixed effects logistic model adjusting for infant’s age (in months) and gender, with robust variances estimators. Random effects were produced at the district level and random intercepts were added at the household level. Each dotted line represents a separate district, while the solid lines represent the average across districts from the same study site

The mixed effects model confirmed that heterogeneity in district-level effects was statistically significant. The predicted probability of full vaccination by year and district are displayed in Fig. 3. In all districts but two, the probability increased between pre- and during pandemic, although the change was not always statistically significant. In only one district was there a significant drop, from 65 to 32%. The heterogeneity in predicted probabilities increased over the period, from 34 to 49% to 33–88% and from 54 to 65% to 32–83% in Sites A and B, respectively.

**Fig. 4:**
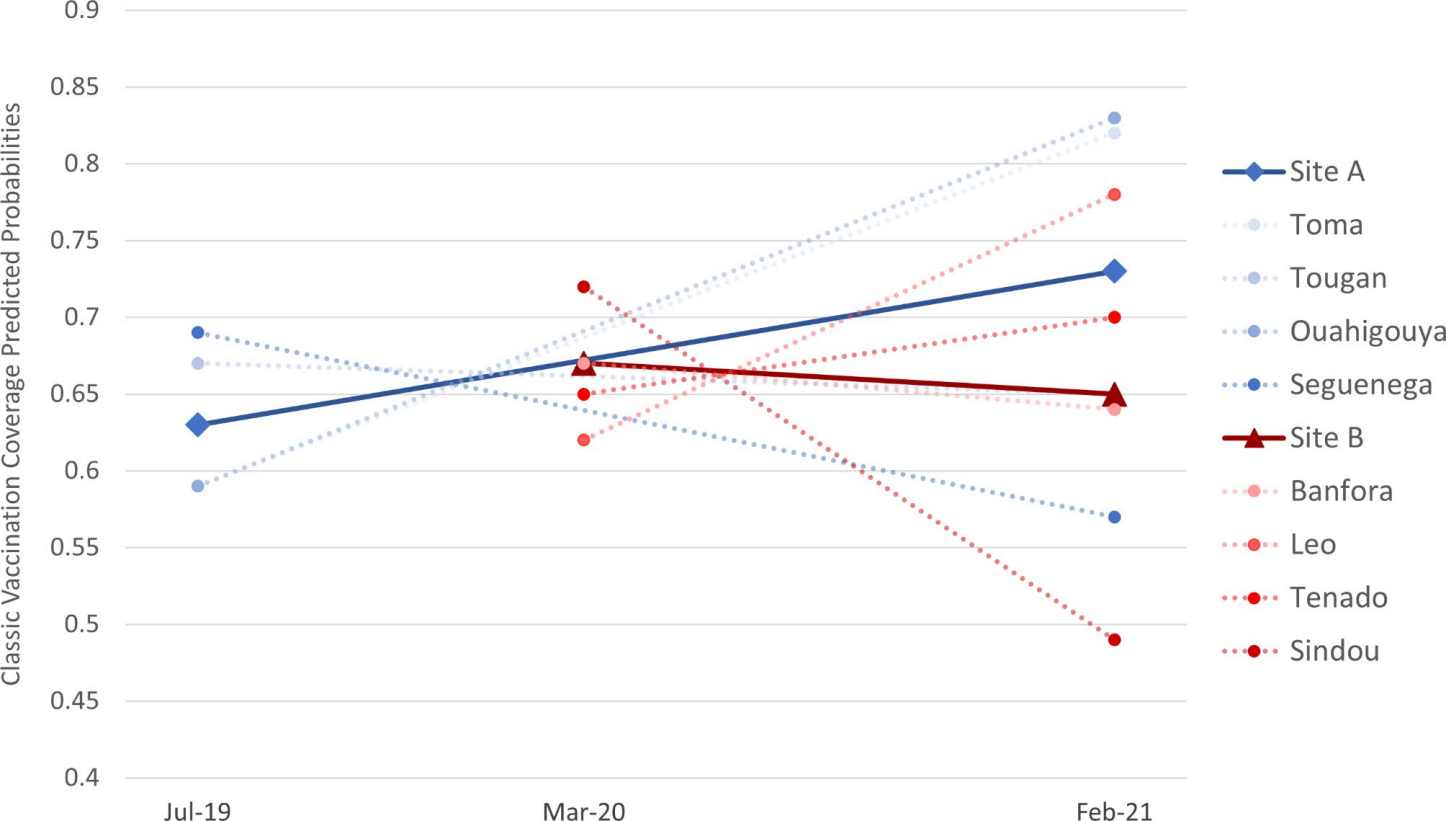
Infant’s predicted probabilities of having age-appropriate full vaccination for the basic vaccines. Predicted probabilities are derived from a mixed effects logistic model adjusting for infant’s age (in months) and gender, with robust variances estimators. Random effects were produced at the district level and random intercepts were added at the household level

When restricting the analyses to the basic vaccines, a positive trend was observed between pre- and during pandemic in Site A, but a negative trend in Site B (Fig. 4). Again, effects vary significantly depending on the district, with half of the districts showing a decrease and the other half an increase. The range in predicted probabilities also increased in both sites, from 59 to 69% to 57–83% for Site A, and from 62 to 72% to 49–78% for Site B.

**Table 2:** Participants’ sociodemographic characteristics

		Pre pandemic n (%)	Per pandemic n (%)	Test of difference (p-value)
N of infants		214	122	
District				0.028
(site A)	Toma	22 (10.7)	7 (5.7)	
	Tougan	26 (12.2)	7 (5.7)	
	Ouahigouya	37 (17.3)	31 (25.4)	
	Seguenega	16 (7.5)	20 (16.4)	
(site B)	Banfora	64 (29.9)	36 (29.5)	
	Sindou	16 (7.5)	6 (4.9)	
	Tenado	14 (6.5)	4 (3.3)	
	Leo	19 (8.9	11 (9.0)	
Residence				0.940
	Urban	86 (40.2)	50 (41.0)	
	Rural	126 (58.9)	72 (59.0)	
	Missing	2 (00.9)	0	
Size of household				0.002
	0–5	40 (18.7)	37 (30.3)	
	6–10	101 (47.2)	63 (51.6)	
	11 and over	73 (34.1)	22 (18.0)	
Socioeconomic status				0.782
	Q1 (poorest)	43 (20.1)	18 (15.1)	
	Q2	37 (17.3)	20 (16.8)	
	Q3	38 (17.8)	20 (16.8)	
	Q4	46 (21.5)	29 (24.4)	
	Q5 (wealthiest)	50 (23.4)	32 (26.9)	
Age of mother (years old)				0.072
	16–25	79 (38.0)	33 (27.7)	
	24–35	96 (46.2)	57 (47.9)	
	36 and above	33 (15.9)	29 (24.4)	
	Missing	6 (2.9)	3 (2.5)	
Education level				0.005
	No education	127 (59.4)	91 (74.6)	
	Primary	34 (15.9)	18 (14.7)	
	Secondary or more	53 (24.8)	13 (10.7)	
Mother has paid job		75 (35.1)	38 (31.2)	0.467
Sex of infant				0.680
	Female	112 (52.3)	61 (50.0)	
	Male	102 (47.7)	61 (50.0)	
Birth order of infant				0.103
	1st	42 (19.6)	17 (14.1)	
	2nd − 3rd	72 (33.6)	43 (35.5)	
	4th − 5th	52 (24.3)	42 (34.7)	
	6th or higher	48 (22.4)	19 (15.7)	
Household radio access		120 (56.1)	71 (58.2)	0.706
Electricity access		67 (31.3)	29 (23.8)	0.141
Bicycle access		176 (82.2)	84 (68.9)	0.000
Infant’s age (months old)				0.103
	0–3	66 (32.0)	34 (27.9)	
	4–6	52 (25.3)	28 (23.0)	
	7–9	33 (15.4)	32 (26.2)	
	10–13	63 (29.4)	28 (23.0)	
Birth setting				0.058
	At home	11 (5.3)	7 (5.7)	
	Public Sector: Hospital	18 (8.4)	20 (16.4)	
	Public sector: Maternity	124 (57.9)	53 (43.4)	
	Public Sector: Health center	56 (26.2)	42 (34.4)	
	Other	5 (2.4)	0	

### Long-term Trends in Vaccination Coverage

Data were extracted from the DHS surveys conducted in the same eight districts in 2010 and 2015. When considering only those children whose vaccination booklet was observed directly, a total of 400 children < 14 months old were added and included in the analyses. The crude average proportion of children with age-appropriate, year-dependent full vaccination reached 58% in 2010, 55% in 2015, and 50% in 2019–2020, suggesting a downward trend before the pandemic.

**Fig. 5:**
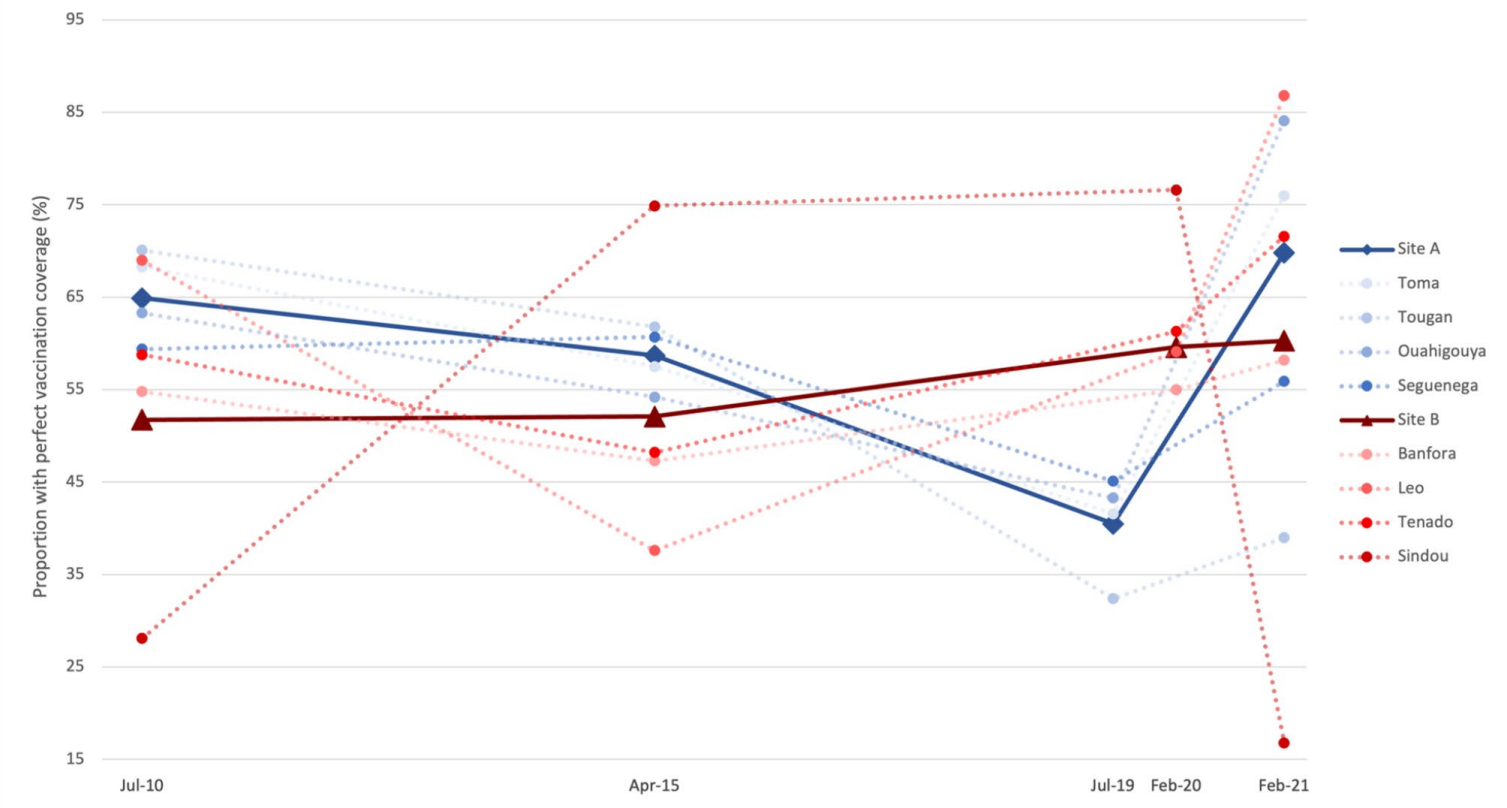
Infants predicted probabilities of having age-appropriate full vaccination from 2010–2021. Predicted probabilities are derived from a mixed effects logistic model adjusting for infant’s age (in months) and gender, with robust variances estimators. Random effects were produced at the district level

**Fig. 6:**
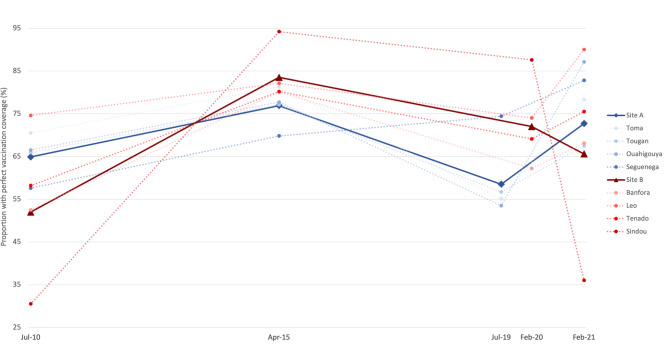
Infants predicted probabilities of having age-appropriate full vaccination for the basic vaccines from 2010–2021. Predicted probabilities are derived from a mixed effects logistic model adjusting for infant’s age (in months) and gender, with robust variances estimators. Random effects were produced at the district level

However, this trend was only observed in Site A, for which the situation considerably improved in 2021. The fixed effects model does not support a hypothesis of significant changes between 2010 and 2021; rather, it suggests an increase in heterogeneity of district responses during the pandemic (Fig. 5). The number of vaccines included in the routine immunization calendar for children < 14 months increased from 9 to 15 between 2010 and 2015 (Table 1). When considering only the basic vaccines, we found less variation across the years and the district responses (Fig. 6).

## Discussion

This study aimed to investigate the effects of the COVID-19 pandemic on age-appropriate full vaccination coverage amongst infants under 14 months of age in rural Burkina Faso, using repeated surveys in a panel of households. Our results showed that on average, coverage increased from 50% before the pandemic to 65% during the pandemic (odds ratio = 1.8, 95% confidence interval [1.14–2.85]). Compared to prior research [29], our study provides a more nuanced and in-depth understanding of the impact of the pandemic on vaccination coverage in rural Burkina Faso. By considering the effects at the district level and including a mixed effects model, our study provides a more comprehensive picture of the effects of the pandemic on vaccination coverage and highlights the importance of considering local context and variation when evaluating public health interventions.

The observed increase in vaccination coverage contrasts with our initial working hypothesis and with previous studies [16–21] that showed a decline in routine immunization rates during the COVID-19 pandemic. Notably, a systematic review of 26 studies conducted in 2022 found that all but five showed significant reductions in immunization coverage during the pandemic [31]. Using a design similar to the one used here, a recent study in Peru found an 8% decrease in the immunization rate amongst infants aged 12–23 months between 2019 and 2021 [32]. Several dynamics likely combined to trigger this phenomenon, including supply difficulties, interruptions in vaccination campaigns, and reduced access to healthcare. In some settings, the COVID-19 pandemic was positively associated with infant vaccination rates: One study found a 35% increase in child vaccination rates in Kenya during the pandemic [33]. Although the reasons are not fully understood, some argue that pandemics can have a positive influence on vaccination coverage due to heightened public awareness and more frequent contact with healthcare services [33, 34]. In Burkina Faso, after the suspension of all door-to-door immunization campaigns on March 27, 2020, a small outbreak of circulating vaccine-derived poliovirus led the authorities to rapidly resume public awareness and mass immunization campaigns in July 2020 [35]. The determination of health authorities to maintain and even strengthen vaccination activities and awareness despite the pandemic may have contributed to an increase of immunization rates. In the present study, the majority of respondents reported feeling little to no threat of contracting COVID-19 through a vaccination program, which may have helped mitigate some negative impacts of the pandemic.

Our study revealed substantial variability in the effects of the pandemic across districts. While some districts experienced a significant increase in vaccination coverage, others observed no change or even a decrease. Our results showed a growing range of predicted probabilities over the period, and the pandemic arguably increased the non-stationarity of the observed trends. Al-kassam-Cordova et al. (2023) reported regional disparities in full-vaccination coverage amongst 12–23-month-old infants in Peru that were related to a variety of geographically distributed factors, including beliefs and customs as well as rugged geography with limited health system access [32]. This was also seen in another study in Burkina Faso showing that children aged 16–36 months living in the Centre Nord, Nord, and Sud-Ouest regions were less likely to be fully vaccinated for their ages than children in other study regions [36]. In our study, only one health district (Sindou) showed a clear downward trend, in contrast to the average trend. This may be an outlier due to its small sample size; there is no indication that this district has encountered any particular difficulties, especially since it is not located in an insecure area [37]. Future research should aim to understand the reasons behind the observed heterogeneity between districts in vaccination coverage. Qualitative research can help identify local contextual factors that may influence vaccination uptake and inform the development of targeted interventions.

Burkina Faso generally has high vaccination coverage compared to other countries in the region, with DTP3 coverage reaching about 90% in infants aged 12–23 months in 2017 [38, 39]; however, in the most recent DHS report, coverage ranged between 56 and 92% from region to region [40]. Previous studies have identified several determinants of infant vaccination coverage in Burkina Faso, including region, distance from health centers, season of children’s birth, rurality, maternal education, and socioeconomic status [36, 41, 42]. Regional disparities in timely vaccination coverage have also been observed, and it has been hypothesized that terrorist attacks are likely to increase these disparities [36, 37].

As suggested by this study and others, the COVID-19 pandemic may also have reinforced existing inequities in vaccination in regards to locality of residence [19, 22, 43, 44]. Arguably, the pandemic has further reduced access to healthcare services in rural and remote areas, whether by reducing door-to-door campaigns and outreach activities, making transport more difficult, causing stock-outs at primary healthcare centers, or increasing home deliveries [45–47]. This heterogeneity highlights the importance of considering local context and variations in public health programs, as well as the local repercussions of natural or external events. To achieve high vaccination coverage rates and protect infants from preventable diseases, it is crucial to prioritize interventions and research efforts to target vulnerable populations who may have been disproportionately affected by the COVID-19 pandemic.

Our study conducted sensitivity analyses to examine the 2010–2021 trends in the proportion of children with age-appropriate full vaccination. The findings do not suggest steady trends, irrespective of the site or the type of vaccines. Coverage increased slightly when from 2010 to 2021, but not steadily; rather, there were upward and downward changes in between. Our trends contrast with those observed in the 2010 and 2021 DHS reports: When we concentrated on basic vaccines, our trends showed an increase in coverage in the Cascades (42.7% vs. 57.1%), Centre-Ouest (65% vs. 86.7%), and Nord regions (64.8% vs. 72.3%), while DHS reports showed heterogenous trends (66.3% vs. 92.9% for Cascades; 82.4% vs. 80.7% for Centre-Ouest; 96.8% vs. 77.4% for Nord) [23, 40]. Poorly organized and inefficient systems, inadequate knowledge amongst health workers about vaccination, and challenges in recalling vaccination dates may have contributed to the decline [48, 49]. The introduction of new vaccines into the Burkina Faso immunization schedule, the rising insecurity and the pandemic may also have played a role [50, 51].

In terms of vaccine coverage, less variation was observed between basic vaccines and those added to the immunization calendar in 2013. The introduction of new vaccines, such as the rotavirus and pneumococcal vaccines, may have contributed to more variability in coverage compared to the basic vaccines. Although the number of vaccines included in the routine immunization calendar for children < 14 months increased from 9 to 15 between 2010 and 2015, the largest declines in vaccination coverage were observed after 2015. One possible reason for this is the challenges in ensuring consistent supply and delivery of these new vaccines after their introduction. Moreover, several countries in West Africa, including Burkina Faso, acquired vaccines from a different source due to global vaccine supply issues in early 2020 [52]. This change, along with the emergence of the pandemic, may have played a role in the variability in vaccination coverage between basic vaccines and newer ones. According to the 2021 DHS report, 79% of children aged 12–23 months received all 8 doses of basic vaccines, while only 36% received all recommended vaccines (basic vaccines plus rotavirus and pneumococcal vaccines) according to the national immunization schedule [40]. This gap between the actual and recommended vaccination coverage is consistent with our results and suggests a need for improvement in the implementation and delivery of vaccination services. Future efforts should include improving vaccine supply chain management and strengthening the capacity of immunization programs to ensure consistent and equitable vaccine access for all individuals.

### Limitations

The biggest limitations of this study are its small sample size and the fact that it was a natural experiment and thus not determined by a power calculation; consequently, some of our estimates have wide confidence intervals. Secondly, our results may not be representative of all the children in the study area, since the analyses were limited to those infants who had a vaccination booklet available to be checked and recorded by surveyors. Excluding infants who did not have a vaccination booklet may have introduced errors in our study; however, sensitivity analyses performed to compare the characteristics of infants with and without vaccination booklets did not indicate any significant differences. This study cannot distinguish between vaccines routinely administered at health facilities and those provided during mass campaigns. Although COVID19 could have affected vaccination coverage through these two mechanisms, it is unlikely that the very brief interruption of mass campaigns could have had a significant impact on vaccination coverage, particularly among infants. Lastly, our study focused primarily on rural children and may not be generalizable to children living in urban areas, since studies have found significant differences in the effects of COVID-19 on vaccination coverage in different settings [32, 36].

## Conclusion

This study provides important information about the effects of the COVID-19 pandemic on infant full-vaccination coverage in rural Burkina Faso. While the pandemic did not significantly affect overall vaccination coverage in the rural districts under study, there was an increase in heterogeneity by district, which may exacerbate existing inequities in health outcomes within Burkina Faso. This highlights the need for continued monitoring and targeted efforts to ensure that all infants receive the full range of recommended vaccinations. Further research is needed to understand the impact of the pandemic on essential health services and to inform effective strategies for maintaining and improving health outcomes during public health emergencies. The study provides valuable insights into trends in vaccination coverage and emphasizes the importance of ongoing monitoring and evaluation of vaccination programs to achieve high coverage rates and equitable health outcomes. Since crises are likely to increase health inequities, health authorities and decision-makers must give priority to strengthening health structures in the most vulnerable regions. Special catch-up vaccination campaigns could also be launched in regions below a certain threshold.

### Electronic supplementary material

Below is the link to the electronic supplementary material.

Supplementary Material 1

## Data Availability

All anonymized data and Stata routine programs can be made available by contacting the corresponding author under reasonable request.

## Abbreviations

DHS: Demographic and Health Survey
DTP3: Diphtheria, tetanus toxoid and pertussis
LMICs: Low- and middle-income countries
WHO: World Health Organization
