# Supplementary material for: Vaccination coverage in rural Burkina Faso under the effects of COVID-19: evidence from a panel study in eight districts
Source: BMC Health Serv Res. 2023 Sep 21;23:1016. doi: 10.1186/s12913-023-10029-1 (PMC10512531; doi:10.1186/s12913-023-10029-1)
Supplement: Supplementary file 1 — Supplementary Material 1 [file 12913_2023_10029_MOESM1_ESM.docx]

Supplementary file 1. Questionnaire (English version)

Source : <https://dhsprogram.com/pubs/pdf/DHSQ6/DHS6_Questionnaires_5Nov2012_DHSQ6.pdf>


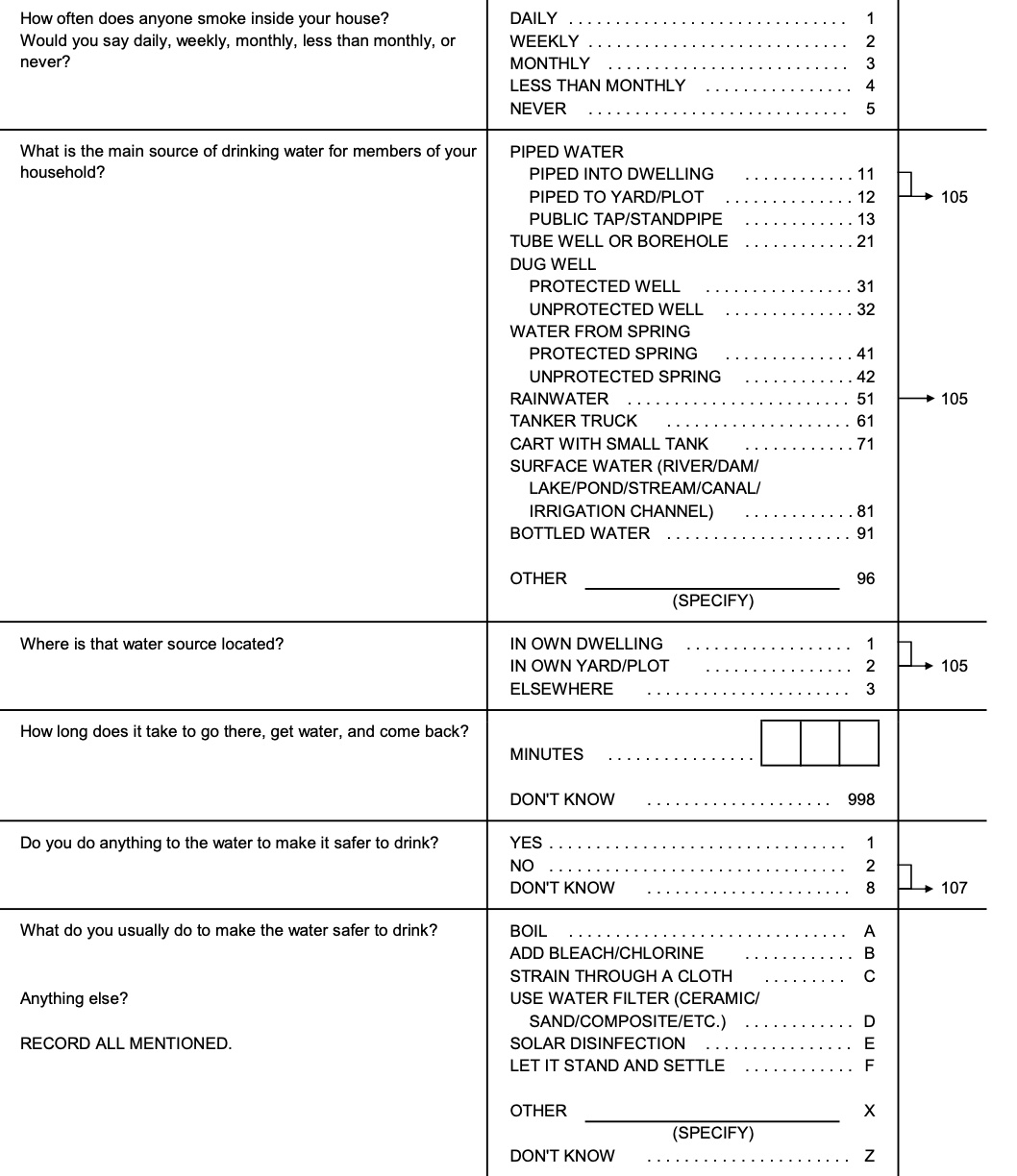


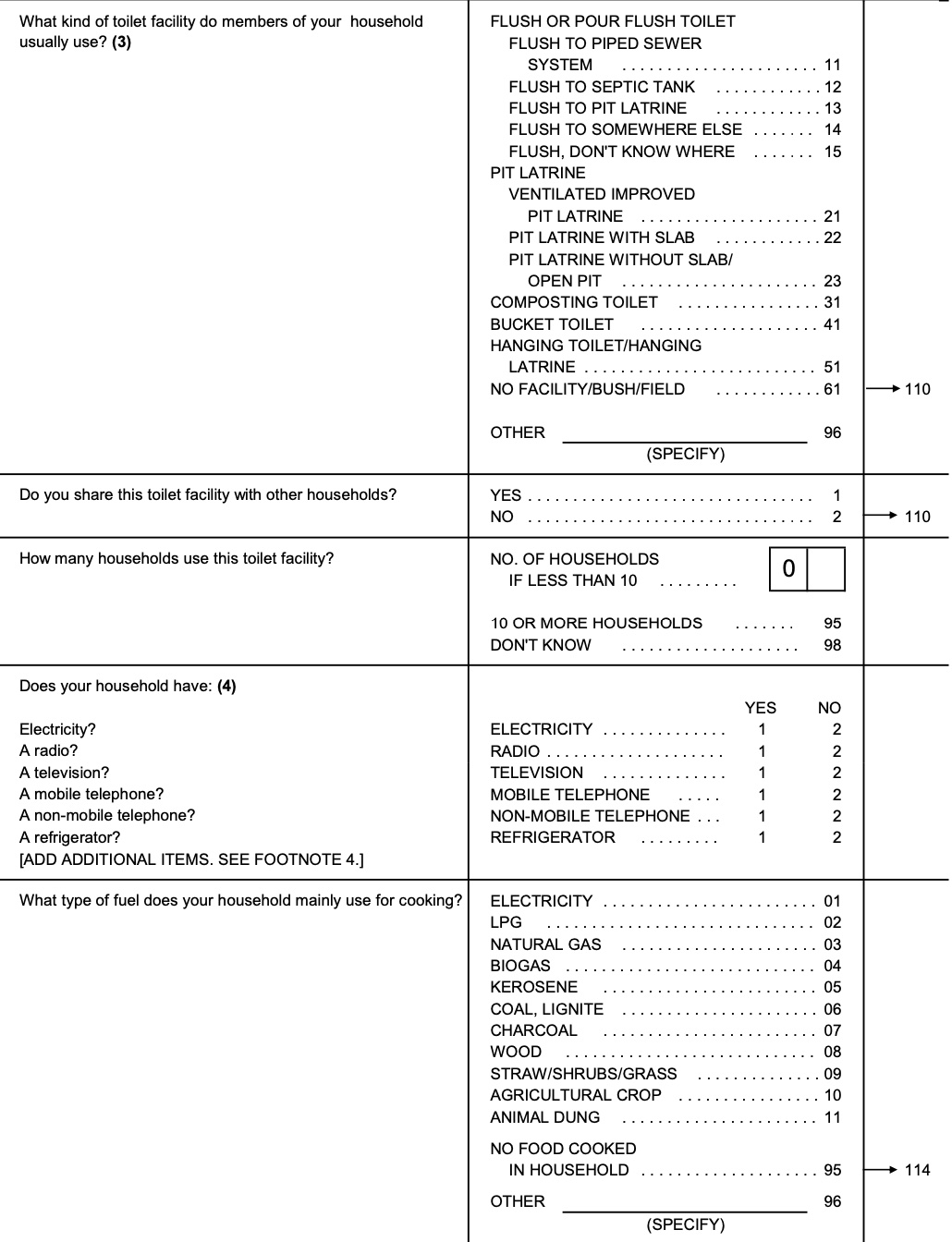


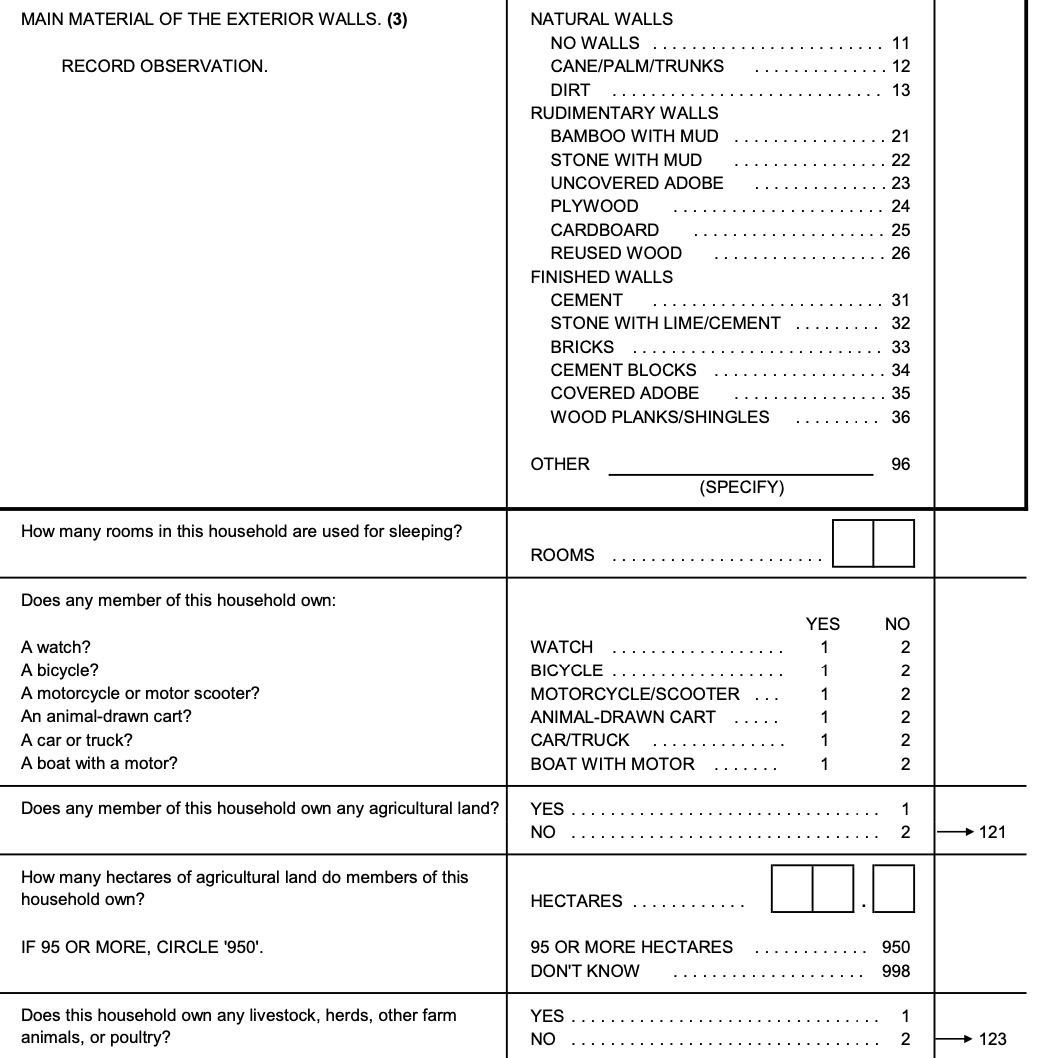


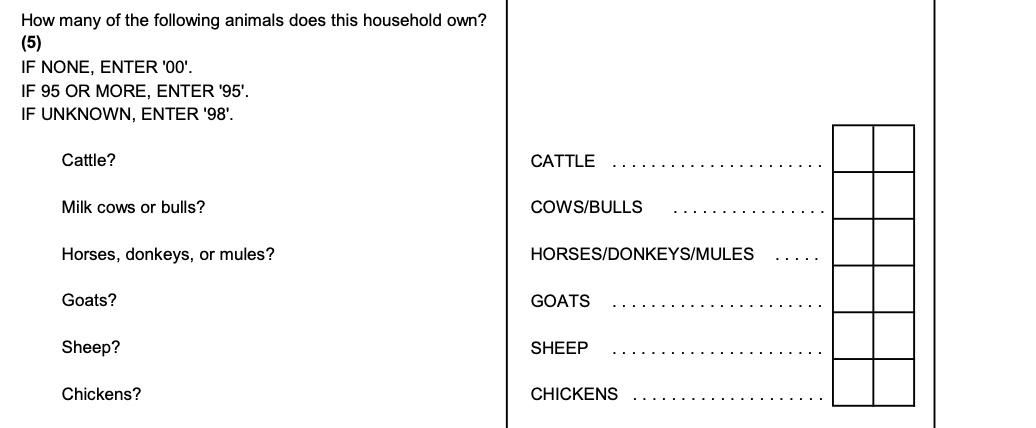


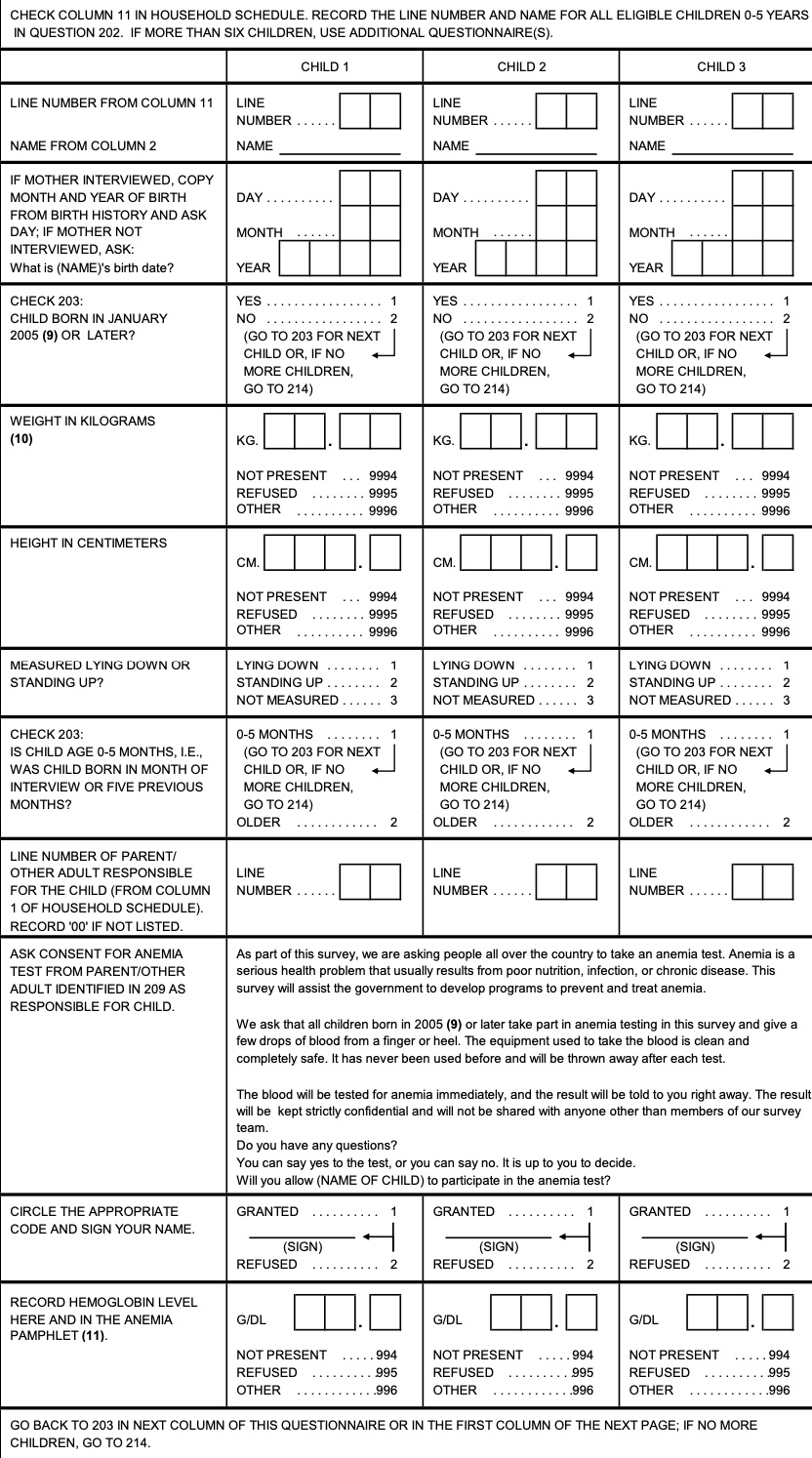


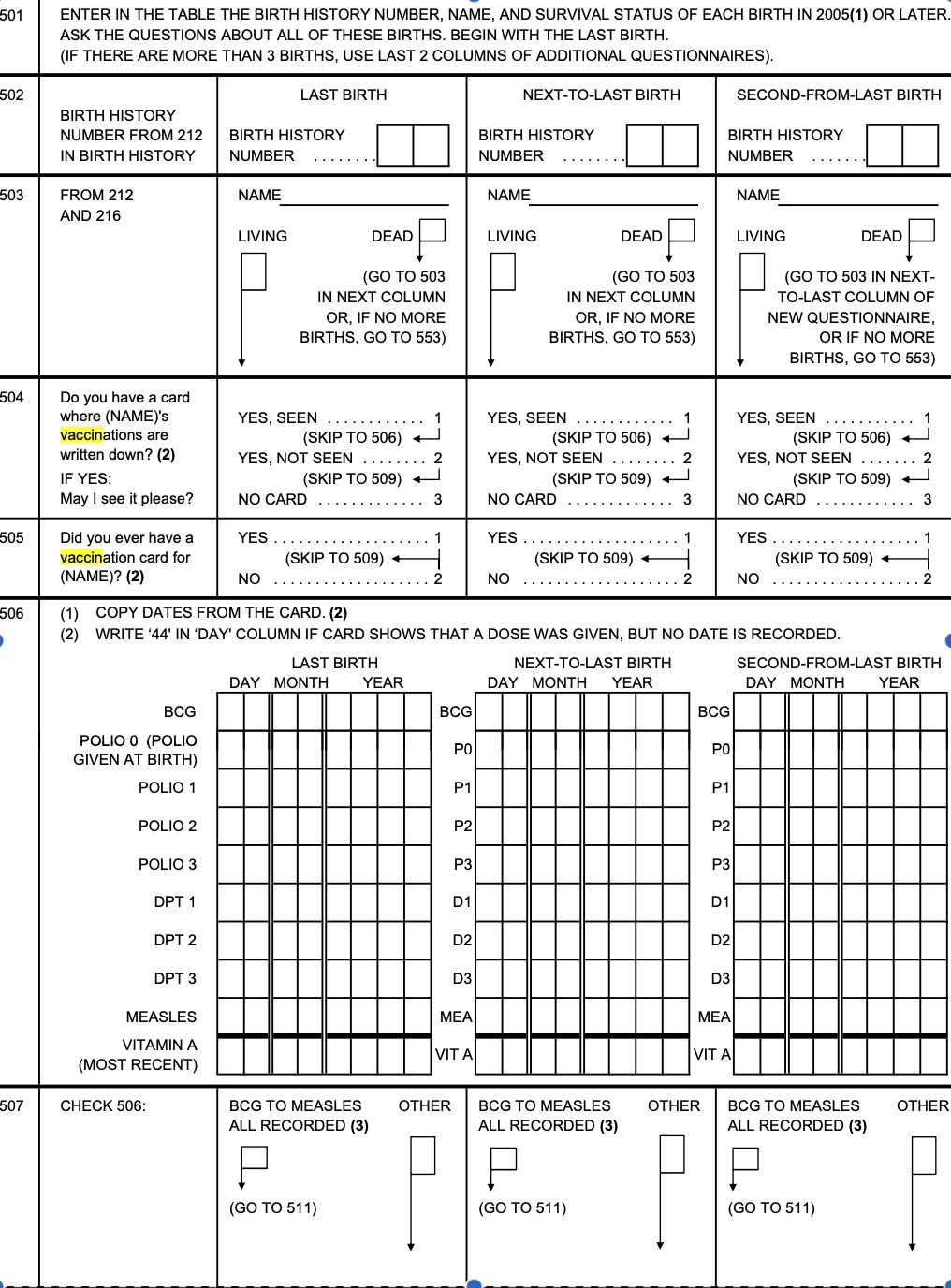


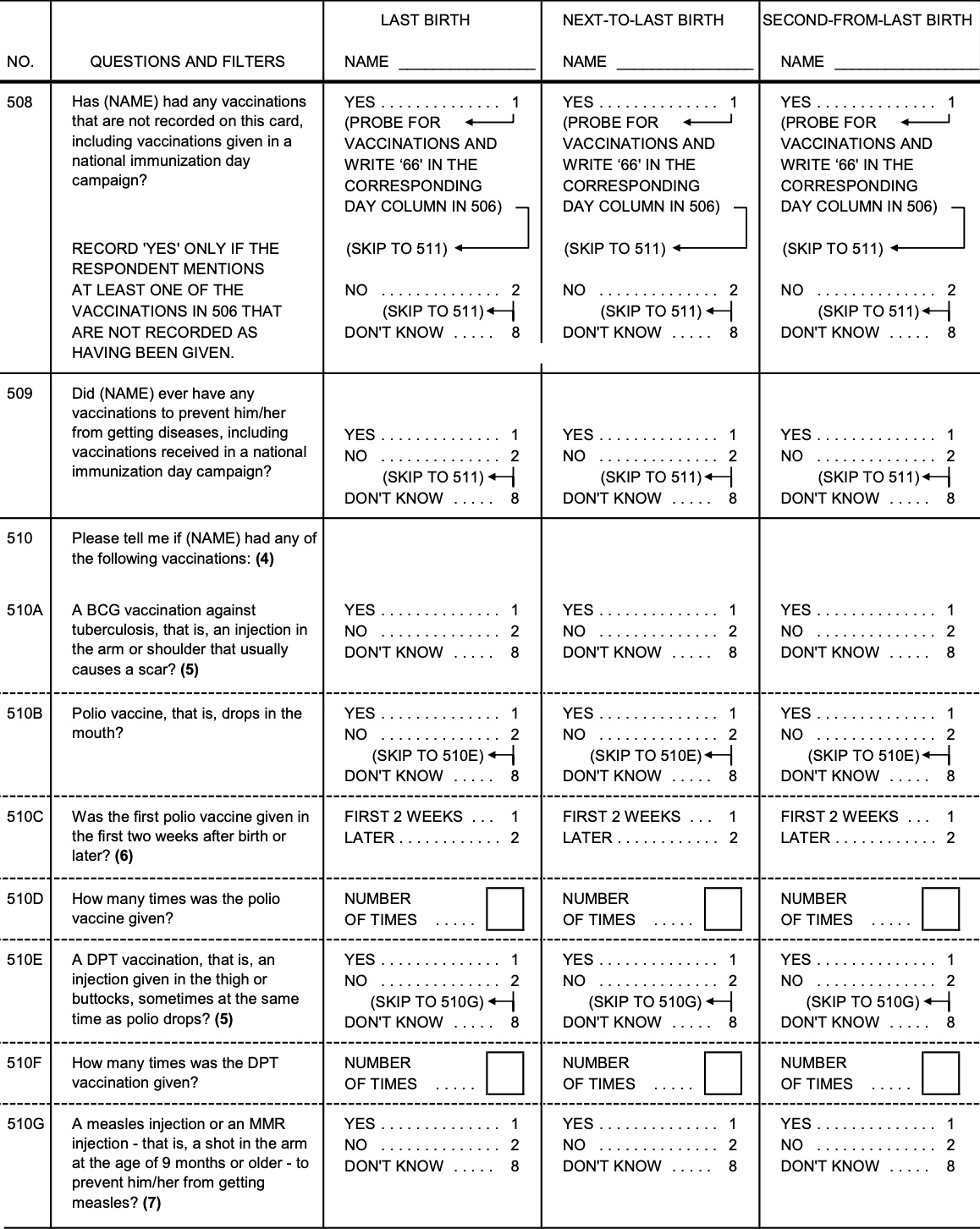


Questionnaire (French version)

Source : <https://dhsprogram.com/pubs/pdf/DHSQ6/DHS6_Questionnaires_French_5Nov2012_DHSQ6.pdf>

## **Module 2. « Informations sur le ménage »**

| 1118 | Est-ce qu’un membre de votre ménage possède :  Une montre ?  Charrues ?  Une bicyclette ?  Un cyclomoteur ?  Une motocyclette ou un scooter ?  Une charrette tirée par un animal ?  Une pirogue/ ou filets de pêche ?  Une voiture ou une camionnette ?  Un bateau à moteur ? | OUI  NON  MONTRE  1 2  CHARRUES 1 2  BICYCLETTE  1 2  CYCLOMOTEUR  1 2  MOTO/SCOOTER 1 2  CHARRETTE  AVEC ANIMAL 1 2  UNE PIROGUE/  FILETS DE PÊCHE ? 1 2  VOITURE/  CAMIONETTE 1 2  BATEAU À MOTEUR 1 2 |
| --- | --- | --- |
| 1119 | Est-ce qu’un membre de votre ménage possède des  terres cultivables ? | OUI = « 1 »  N0N = « 2 » |
| 1121 | Est-ce que votre ménage possède du bétail, des troupeaux, d’autres animaux de ferme ou de la volaille? | OUI = « 1 »  NON = « 2 » |
| 1122 | Parmi les animaux suivants, combien votre ménage en possède-t-il ?  SI AUCUN, INSCRIVEZ « 0 »  SI 95 OU PLUS, INSCRIVEZ « 95 »  SI NE SAIT PAS, INSCRIVEZ « 998 »  Bétail ?  Vaches laitières ou taureaux ?  Chevaux, chameaux, ânes ou mules ?  Chèvres ?  Moutons ?  Volailles ? | BÉTAIL = ____  VACHES/TAUREAUX = ____  CHEVEAUX/CHAMEAUX/  ÂNES/MÛLES = ____  CHÈVRES = ____  MOUTONS = _____  VOLAILLES = _____ |
| 1123 A | Est-ce que quelqu’un de votre ménage a reçu un transfert d’argent au cours des 12 derniers mois ? | OUI = « 1 »  NON = « 2 » |

## ****Module 3 : Questionnaire « Femme »****

### Volet 1. Caractéristiques sociodémographiques de l’enquête

| NUMÉRO | QUESTIONS ET FILTRES | CODES |
| --- | --- | --- |
| 110 | Enregistrez l’heure | HEURE : ____ ____  MINUTES : ____ ____ |
| 111 | En quel mois et en quelle année êtes-vous née ? | MOIS : ___________  NE CONNAÎT PAS LE MOIS = « 998 »  ANNÉE : ___________  NE CONNAÎT PAS L'ANNÉE = « 9998 » |
| 112 | Quel âge aviez-vous à votre dernier anniversaire ?  COMPAREZ ET CORRIGEZ Q. 111 ET/OU 112 SI INCOHÉRENT. | ÂGE EN ANNÉES  RÉVOLUES : ______ |
| 113 | Êtes – vous allée à l’école ? | OUI = « 1 »  NON = « 2 » |
| 114 | Quel est le plus haut niveau d’études que vous avez atteint :  primaire, secondaire 1 (premier cycle), secondaire 2 (deuxième cycle) ou supérieur ? | PRIMAIRE = « 1 »  SECONDAIRE (1^ER^ CYCLE) = « 2 »  SECONDAIRE (2^ÈME^ CYCLE) = « 3 »  SUPÉRIEUR = « 4 » |

###

### Volet 2. « Naissances »

| 201 | Je voudrais maintenant vous poser des questions  sur toutes les naissances que vous avez eues  durant votre vie. Avez-vous déjà  donné naissance à des enfants ?  Mentionnez que ceci implique les naissances d’enfants vivants, même si l’enfant est décédé ensuite. Les naissances d’enfants mort-nés ne sont pas comptabilisées. Les fausses couches et avortements ne sont pas comptabilisés ici. | OUI = « 1 »  NON = « 2 » |
| --- | --- | --- |
| 203 | À combien de fils avez-vous donnée naissance ?  Et combien de filles?  SI AUCUN, INSCRIVEZ « 0 » | FILS À LA MAISON : ______  FILLES À LA MAISON : ______ |
| 206 | Avez-vous déjà donné naissance à un garçon ou à une fille qui est né vivant mais qui est décédé par la suite ? | OUI = « 1 »  NON = « 2 » |
| 207 | Combien de garçons sont décédés ?  Combien de filles sont décédés ?  SI AUCUN, INSCRIVEZ « 0 » | GARÇONS DÉCÉDÉS : ______  FILLES DÉCÉDÉES : ______ |
| 208 | Programmer pour calcul |  |
| 209 | VÉRIFIEZ Q.208:  Je voudrais être sûre d'avoir bien compris :  vous avez eu au TOTAL_____ naissances vivantes durant votre vie.  Est-ce bien exact ? | TOTAL NAISSANCES Q. 39 : _____ ;  OUI : « 1 », SI TOTAL NAISSANCE(S) Q.39 =TOTAL NAISSANCE(S) Q. 38  NON : « 2 », SI TOTALNAISSANCE(S) Q.39 ≠TOTAL NAISSANCE(S) Q. 38 |
| 212 | Quel nom a été donné à votre  (premier enfant/ enfant suivant) ? | NOM : ___________ |
| 213 | (NOM) EST-IL UN GARÇON OU UNE FILLE ? | GARÇON = « 1 »  FILLE = « 2 » |
| 215 | En quel mois et en quelle année est né (NOM)  INSISTEZ : Quelle est sa date de naissance ? | MOIS : _______  ANNÉE : _______ |
| 216 | (NOM) est-il/elle encore en vie ? | OUI = « 1 »  NON = « 2 » |
| 218 | SI EN VIE :  (NOM) vit- il/elle avec vous ? | OUI = « 1 »  NON = « 2 » |
| 220 | SI DÉCÉDÉ :  QUEL ÂGE AVAIT (NOM) QUAND IL/ELLE EST DÉCÉDÉ(E) ?  SI « 1 AN », INSISTEZ :  Combien de mois avait (NOM) ?  INSCRIVEZ EN JOURS SI MOINS D’1 MOIS  EN MOIS SI MOINS DE 2 ANS ;  OU EN ANNÉES. | JOURS  « 1 »  = ___________  MOIS « 2 » = ____________  ANNÉES « 3 » = __________ |
| 226 | Êtes-vous actuellement enceinte ? | OUI = « 1 »  NON = « 2 »  NE SAIT PAS = « 998 » |
| 227  C | Depuis combien de mois êtes-vous enceinte ?  ENREGISTREZ LE NOMBRE DE MOIS RÉVOLUS. | MOIS = ________ |
| 228 | Quand vous êtes tombée enceinte,  vouliez- vous être enceinte à ce moment-là ? | OUI = « 1 »  NON = « 2 » |
| 229 | Est-ce que vous vouliez avoir un enfant plus tard  ou est-ce que vous ne vouliez pas/plus d'enfant ? | PLUS TARD : « 1 »  NE PAS/NE PLUS  AVOIR D’ENFANT : « 2 » |

| 230 | Avez-vous déjà eu une grossesse qui s'est terminée  par une fausse couche, un avortement  ou un mort-né ? | OUI = « 1 »  NON = « 2 » |
| --- | --- | --- |
| 231 | Quand la dernière grossesse de ce genre  s'est-elle terminée ? | MOIS = ________  ANNÉE = _______ |
| 232 | VÉRIFIEZ Q. 231  DERNIÈRE GROSSESSE TERMINÉE : | EN JANVIER 2014 OU  PLUS TARD = « 1 »  DERNIÈRE GROSSESSE TERMINÉE AVANT JAN. 2014 = « 2 » |
| 233  C | De combien de mois étiez-vous enceinte quand la dernière grossesse de ce genre s’est terminée ?  INSCRIVEZ LE NOMBRE DE MOIS RÉVOLUS.  INSCRIVEZ « F » DANS LE CALENDRIER AU MOIS OU LA GROSSESSE S’EST TERMINÉE ET « G » POUR LE NOMBRE RESTANT DE MOIS RÉVOLUS. | MOIS = _______ |
| 234 | Depuis janvier 2020, avez-vous eu d'autres grossesses  qui n'ont pas abouti à une naissance vivante ? | OUI = « 1 »  NON = « 2 » |
| 236 | Avez-vous eu une grossesse qui a pris fin avant 2020  et qui s'est terminée par une fausse-couche,  un avortement ou un mort-né ? | OUI = « 1 »  NON = « 2 » |
| 237 | Quand la dernière grossesse de ce genre  s'est-elle terminée avant 2020 ? | MOIS = ________  ANNÉE = _______ |

### Volet 5. « Vaccinations des enfants, santé et nutrition »

| 502 | NUMÉRO DE LIGNE Q.212  DANS L’HISTORIQUE DES NAISSANCES | DERNIÈRE NAISSANCE  NUMÉRO HISTORIQUE  NAIS. : ________ |
| --- | --- | --- |
| 503 | À PARTIR DES QUESTIONS 42 À 46 | NOM : _________  VIVANT = « 1 »  DÉCÉDÉ = « 2 »  *SI = « 2 » → Q.503 À LA COLONNE (NAISSANCE) SUIVANTE, OU SI IL N’Y A  PLUS DE NAISSANCE, ALLEZ  À Q. 553 |
| 504 | Avez-vous un carnet où  les vaccinations de  (NOM) sont inscrites ? | OUI, VU= « 1 » →Q.506  OUI, PAS VU = « 2 » →Q.509  PAS DE CARNET = « 3 » |

| 507 | VÉRIFIEZ Q. 506 | BCG À ROUGEOLE TOUT ENREGISTRÉ = « 1 »  AUTRE = « 997 »  *SI = « 1 » → Q.511 |
| --- | --- | --- |
| 508 | (NOM) a-t-il/elle eu des vaccins qui  ne sont pas inscrits sur le carnet, y  compris des vaccins reçus le jour  d'une campagne nationale de  vaccination ? | OUI = « 1 »  (INSISTEZ POUR LES  VACCINS ET INSCRIVEZ  « 66 » À LA COLONNE  CORRESPONDANT  AU JOUR À Q. 506)  NON = « 2 »  NE SAIT PAS = « 998 »  *SI = « 1 », « 2 » OU « 998 »→ Q. 511 |
| 509 | (NOM) a t-il/elle déjà eu des  vaccins pour lui éviter de contracter  des maladies, y compris des  vaccins reçus le jour d'une  campagne nationale de  vaccination ? | OUI = « 1 »  NON = « 2 »  NE SAIT PAS = « 998 »  SI = « 2 » OU «  998 »  → Q. 511 |
| 510  510 A | Dites-moi si (NOM) a eu l’un des vaccins  Suivants :  Le vaccin du BCG contre la tuberculose,  c’est-à-dire une injection dans le bras  ou à l’épaule qui laisse habituellement  une cicatrice ? | OUI = « 1 »  NON = « 2 »  NE SAIT PAS = « 998 » |
| 510 B | Le vaccin de la polio, c'est-à-dire  des gouttes dans la bouche ? | OUI = « 1 »  NON = « 2 »  NE SAIT PAS = « 998 »  SI = « 2 » OU «  998 »  → Q. 510 E |
| 510 C | Le premier vaccin de la polio a-t-il  été donné dans les 2 premières  semaines après la naissance ou  plus tard ? | 2 1^ÈRE^ SEMAINES = « 1 »  PLUS TARD = « 2 » |
| 510 D | Combien de fois le vaccin de la polio a-t-il été  donné | NOMBRE DE FOIS = _____ |

| 510 E | Le vaccin du DTCoq-HépB+Hib,  c'est-à-dire une injection faite à la  cuisse ou à la fesse, parfois donné  en même temps que les gouttes  pour la polio ? | OUI = « 1 »  NON = « 2 »  NE SAIT PAS = « 998 »  *SI = « 2 » OU « 998 »  → Q.511 |
| --- | --- | --- |
| 510 F | Combien de fois le vaccin du  DTCoq a-t-il été donné ? | NOMBRE DE FOIS = _____ |
| 510 G | Le vaccin contre la rougeole ou le  ROR, c'est-à-dire une injection  dans le bras à l'âge de 9 mois ou  plus tard, pour lui éviter la  rougeole ? | OUI = « 1 »  NON = « 2 »  NE SAIT PAS = « 998 » |
